# Supplementary material for: MetaRibo-Seq measures translation in microbiomes
Source: Nat Commun. 2020 Jun 29;11:3268. doi: 10.1038/s41467-020-17081-z (PMC7324362; doi:10.1038/s41467-020-17081-z)
Supplement: Supplementary file 10 — Supplementary Data 7 [file 41467_2020_17081_MOESM10_ESM.zip › File2/Confidence_VeryHigh_Taxonomy/419212_out.krona.html]

Javascript must be enabled to view this page.

members
magnitude
magnitudeUnassigned
count
unassigned
taxon
rank

419212\_out

4

2
4
superkingdom

1239
phylum
4

186801
class
4

186802
1
order
4

SRS104485\_contig\_number\_contig-100\_15622.15622

216572
family
1

459786
1
genus

876091
species
1

SRS1041090\_contig\_number\_754

31979
1
family

genus
1
580596

species
1

SRS077231\_contig\_number\_contig-100\_2461.139948
2292295

1
family
541000

1
genus
1929297

1
species

SRS147653\_contig\_number\_7335
1720313
